# Sarcomatoid Carcinoma of the Oral Cavity: A Diagnostic Dilemma

**DOI:** 10.1155/2017/7495695

**Published:** 2017-12-17

**Authors:** Anshul Mahajan, Sujata Mohanty, Sujoy Ghosh, Aadithya B. Urs, Nita Khurana, Sunita Gupta

**Affiliations:** ^1^Department of Oral Medicine & Radiology, Maulana Azad Institute of Dental Sciences, New Delhi, India; ^2^Department of Oral & Maxillofacial Surgery, Maulana Azad Institute of Dental Sciences, New Delhi, India; ^3^Department of Oral Pathology, Maulana Azad Institute of Dental Sciences, New Delhi, India; ^4^Department of Pathology, Maulana Azad Medical College, New Delhi, India

## Abstract

Sarcomatoid carcinoma (SC) is a rare variant of squamous cell carcinoma which is characterized by a dysplastic epithelial component and a stromal element with invasive fusiform or spindle-shaped cells. The clinical and histopathologic characteristics make it very difficult to distinguish SC from epithelioid sarcoma (ES). We present a case of a 51-year-old man with a soft tissue mass in the oral cavity diagnosed as proximal variant of epithelioid sarcoma on incisional biopsy. A thorough radiologic examination was conducted to rule out the possibility of a primary elsewhere in the body. Supraomohyoid neck dissection, mandibular resection, and reconstruction with recon plates were carried out. Histopathologic examination was suggestive of epithelioid variant of SC which was contrary to the incisional biopsy report. The dilemma in diagnosis was resolved by observing the presence of invading atypical epithelial cells into the stroma confirming the epithelial origin of the tumor.

## 1. Introduction

Sarcomatoid carcinoma (SC), also known as spindle cell carcinoma (SpCC) and polypoid squamous cell carcinoma, is a rare variant of squamous cell carcinoma characterized by dysplastic surface squamous epithelium along with an invasive spindle cell element [1]. It shows a wide range of age of occurrence with definite male predilection [2, 3]. Clinical presentation is mostly exophytic and polypoid, but nodular or endophytic appearance has also been described [2]. SC shows a biphasic histologic appearance with epithelial changes varying from dysplasia to invasive carcinoma and stromal component composed of fusiform or spindle-shaped cells [2–4]. This histologic appearance makes it a challenge for the pathologist to arrive at a diagnosis. We share a similar dilemma of diagnosing a case of sarcomatoid carcinoma of the oral cavity in a 51-year-old man previously diagnosed with epithelioid sarcoma (ES) discussing the histopathologic aspects that differentiate them.

## 2. Case Report

A 51-year-old man reported to the department with a complaint of soft tissue growth in the mandibular left second and third molar region since about 15 days. The growth was small when he first noticed it and was associated with mobility of mandibular left third molar. He showed to a local dentist who extracted the tooth with excision of the mass. No histopathological examination was conducted on the excised tissue mass. Post extraction, the growth rapidly increased in size to reach the current size. Past medical history was insignificant. The patient also did not present with any habit of smoking and tobacco or alcohol consumption. General physical examination was conducted which revealed an otherwise healthy individual with a short and thin built, normal gait, and no history of any fever, headache, or weight loss in the recent past. The left submandibular lymph nodes were enlarged, tender, and fixed to the underlying tissues.

**Figure 1:**
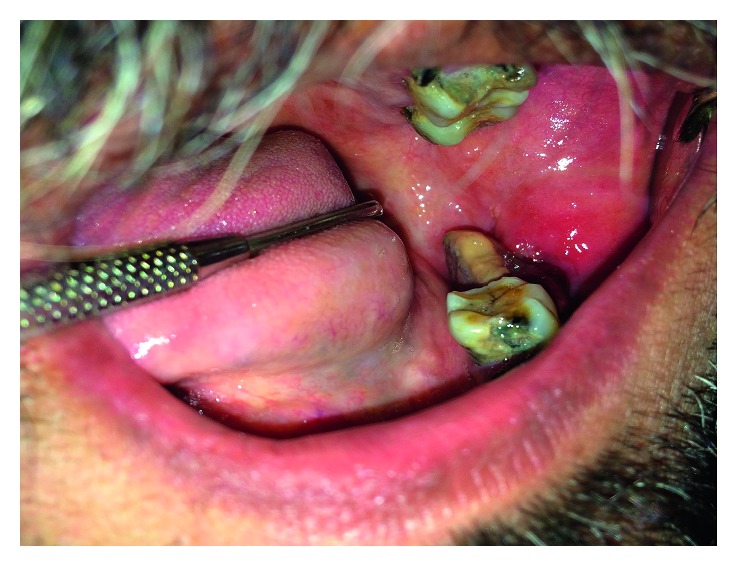
Clinical presentation. Intraoral examination showed a reddish white, lobular, soft gingival mass on the left mandibular alveolar ridge in the region of mandibular left third molar.

Intraoral examination revealed a 2.5 cm × 2 cm irregularly shaped, reddish-white, lobular, soft gingival mass on the left mandibular alveolar ridge in the region of mandibular left third molar which was tender on palpation and occasionally bled. There was no ulceration or surface erosion (Figure 1). A detailed hard tissue examination revealed a poor dental hygiene with multiple root stumps and decayed teeth. Routine hematological tests were conducted which were within the normal range except ESR which was elevated. Panoramic radiograph revealed a well-defined arc-shaped osteolytic lesion with noncorticated borders extending from the distal aspect of mandibular left first molar to anterior border of ascending ramus.

**Figure 2:**
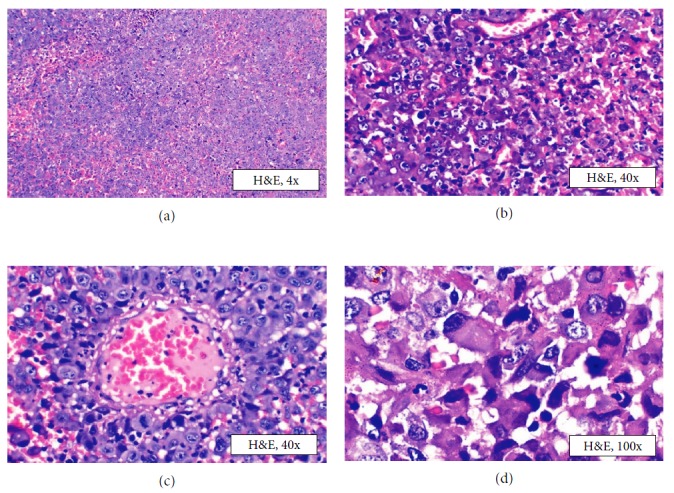
Incisional biopsy. Photomicrograph of incisional biopsy tissue showing (a) hypercellular lesional tissue proliferating in sheets with hemorrhagic background, 4x H&E; (b) pleomorphic epithelioid cells with vesicular nucleus, prominent nucleoli, 40x H&E; (c) tumor cells radiating out from blood vessel in streaming fashion (pleomorphism and atypical mitoses are also seen), 40x H&E; (d) tumor cells with rhabdoid cells appearance and pleomorphism, 100x H&E.

**Figure 3:**
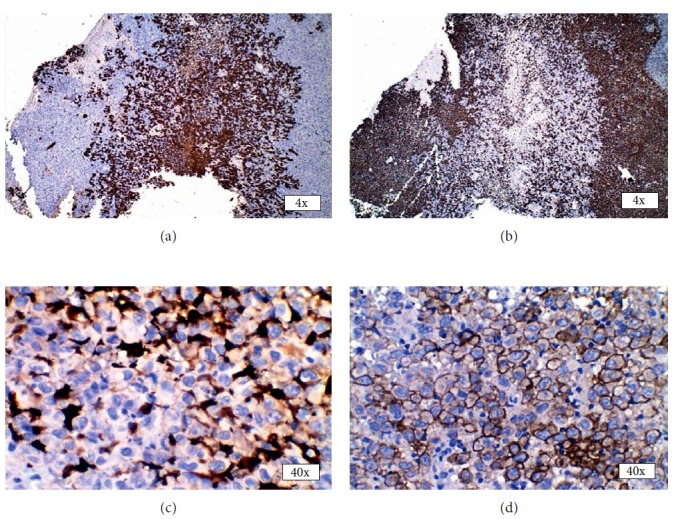
Immunohistochemistry. Immunohistochemical markers showing positive cytokeratin (a), vimentin (b), epithelial membrane antigen (d), and negative S100 (c).

**Figure 4:**
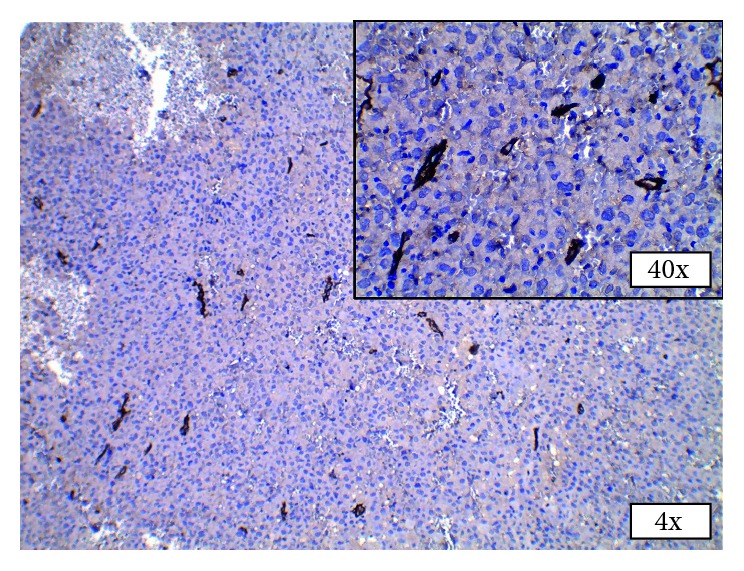
Immunohistochemistry CD34. Tumor cells negative for CD34, 4x, and 40x (inset).

The gingival growth was biopsied under local anesthesia, the findings of which were suggestive of proximal variant of epithelioid sarcoma (ES) (Figures 2–2). Immunohistochemistry performed on the tissue showed diffuse strong cytoplasmic positivity for pancytokeratin and vimentin (Figures 3 and 3). EMA was strongly positive with membranous staining of the tumor cells in >75% of the tumor cell population (Figure 3). All other markers of S100 (Figure 3), desmin, CD45, CD31, and CD34 (Figure 4) were negative for the tumor cells.

**Figure 5:**
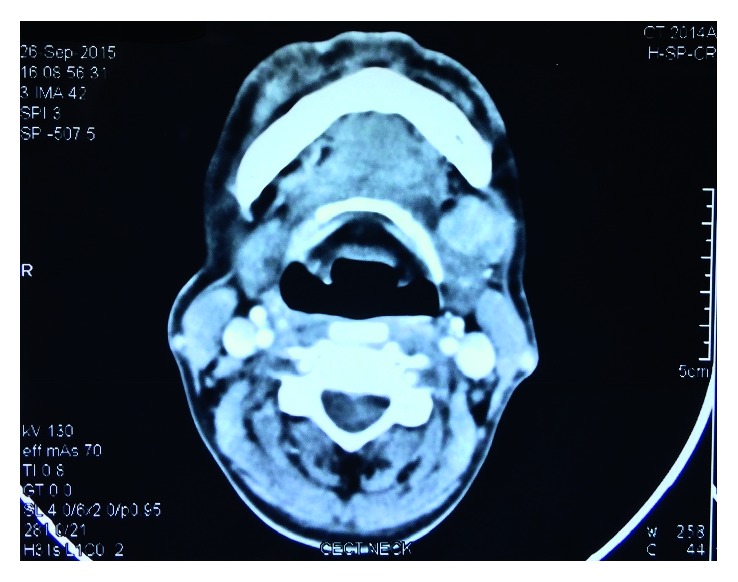
Contrast-enhanced computed tomography. CECT axial section shows ill-defined heterogeneously enhancing soft tissue lesion in the left submandibular region with no obvious bone erosion. Few subcentimetric nodes are seen along bilateral submandibular and upper jugular chains.

**Figure 6:**
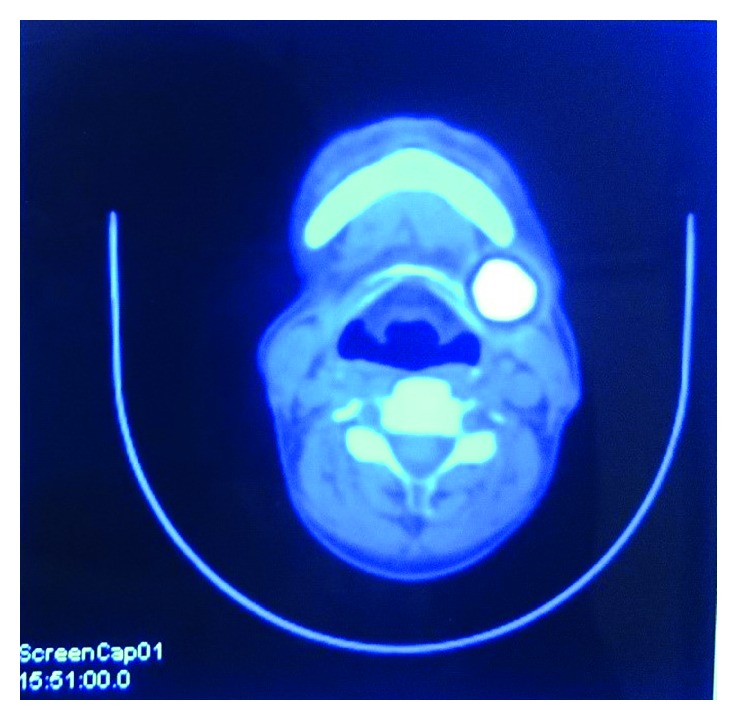
Positron emission tomography. ^18^F-FDG PET-CT axial section shows hypermetabolic lesion involving the region of mandibular left third molar and level IB cervical lymph node.

To rule out the possibility of a primary elsewhere in the body, advanced imaging modalities like ultrasound abdomen, contrast enhanced CT (CECT) of head and neck region, and positron emission tomography (PET) scan were carried out, all of which revealed the gingival growth to be the primary lesion (Figures 5 and 6). A surgical approach to management was considered as appropriate which included supraomohyoid neck dissection (levels IA, IB, IIA, IIB, and III), excision of submandibular gland, and tail of parotid gland under general anesthesia. Mandible was exposed and resected till mandibular left premolar region along with the soft tissue growth with 1.5 cm safe margin. A recon plate was admitted and fixed using three 2.5 × 10 mm screws. Negative margins were confirmed using the frozen section. Following the surgery, the patient was subjected to chemotherapy.

**Figure 7:**
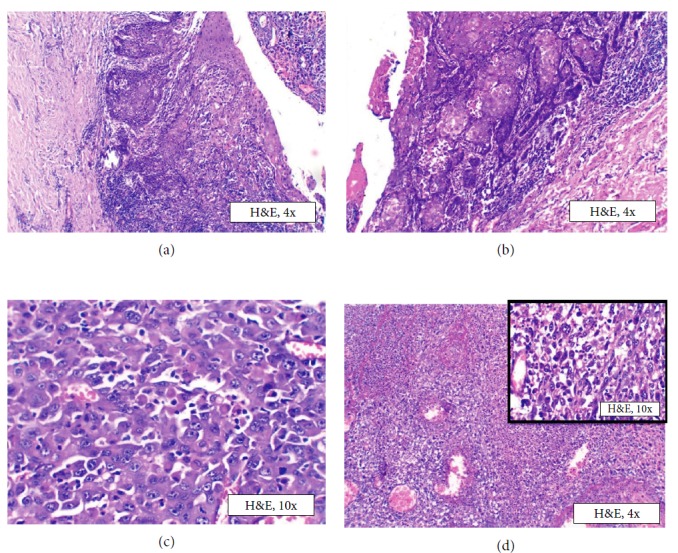
Excisional biopsy. Photomicrograph of excisional biopsy showing (a, b) dysplastic epithelium proliferating into connective tissue, 4x H&E; (c) pleomorphic cells with vesicular nucleus, rhabdoid appearance, 10x H&E; (d) sheets of biphasic tumor cells with spindle cells in inset, 10x H&E.

Histopathologic examination following excision revealed overlying parakeratinized stratified squamous epithelium at either end with ulceration and discontinuity at the centre (Figures 7 and 7). The ulcerated area showed abundant epithelioid cells in loosely held stroma showing highly dysplastic features of pleomorphism, altered nucleocytoplasmic ratio, and atypical mitoses (Figure 7). At areas, the adjacent epithelium was showing dysplastic features with invasion of these cells into the stroma along with the epithelioid cells. This was associated with a dense chronic inflammatory cell infiltrate. The epithelioid cells were highly undifferentiated and admixed with few spindle-shaped cells with mitotic figures (Figure 7). Abundant rhabdoid cells with typical eccentric nuclei and cytoplasmic inclusions were seen scattered throughout. Invasion of the tumor cells into blood vessels and underlying muscle was also seen. The final diagnosis for the excisional tissue was determined as epithelioid variant of SC since the epithelioid component predominated over the spindle cells. The dilemma in diagnosis was resolved because the invasion of the overlying atypical epithelial cells into the stroma was clearly evident in the final tissue received and hence confirming the epithelial origin of the tumor.

## 3. Discussion

Sarcomatoid carcinoma (SC) is a rare variant of squamous cell carcinoma characterized by dysplastic surface squamous epithelium along with an invasive spindle cell element [1]. Different authors have different views regarding histogenesis of SC and have used various terms to describe it. Virchow in 1864 first reported it and labeled it as carcinosarcoma, suggesting that it may be a “collision tumor” between a carcinoma and sarcoma [1–3]. Krompecher in 1900 proposed an epithelial origin with “dedifferentiation” to a spindle cell morphology and used the term “sarcomatoid carcinoma” to describe it [2, 3]. Lane in 1957 proposed the term “pseudocarcinoma” suggesting that it may be a squamous cell carcinoma with an atypical reactive stroma [2, 3]. This multiplicity in nomenclature indicates the complexity of its histogenesis.

Epithelioid sarcoma (ES) on the other hand is a soft tissue tumor composed of large polygonal cells resembling carcinomas [5]. It is rare in occurrence (<1% of all soft tissue sarcomas) with unknown histogenesis and seemingly benign pathomorphologic appearance and is hence often misdiagnosed on first encounter. ES is a mesenchymal tumor with a predominant epithelial differentiation showing reactivity for both epithelial and mesenchymal markers [5]. This similarity in histological features of the two entities poses a great dilemma to the clinician and pathologist in establishing a final diagnosis.

Sarcomatoid carcinoma of the oral cavity comprises less than 1% of all tumors of the oral cavity [3]. It has a wide age of occurrence ranging from 2nd to 9th decade and a mean age during the 5th decade with a predominant male predilection [3, 4]. Although most tumors in the head and neck region occur in the larynx, in the oral cavity, it has a site predilection for the lower lip, tongue, and alveolar ridge or gingiva [2, 6]. Vishwanathan et al. in their study of 103 cases of SC reported an incidence of 17.5% in the larynx and 63.1% in the oral cavity [4]. In the larynx, true cords and the supraglottic areas are predominant sites of occurrence with the subglottic area being an unusual location [6]. Pyriform sinus is the preferred site in pharynx, as are nasal cavity and maxillary antrum in the sinonasal tract [6].

Clinically, SC most commonly presents as a painful swelling or a nonhealing ulcer [3]. The growth configuration is often exophytic polypoid, but sessile, nodular, or endophytic configuration has also been described. The lesion usually has an extensive surface ulceration with friable, fibrinoid necrosis of variable thickness or shaggy exudates [2]. Occasionally, bits of the tumors appear in expectorations [6]. Radiation, trauma, tobacco use, and alcohol consumption seem to play a role as etiological factors [2]. These factors were all negative in the present case.

SC shows a biphasic histologic appearance with surface epithelium showing features of mild dysplasia to invasive carcinoma and an atypical stroma composed of fusiform cells giving a fibrosarcoma-like appearance [3, 6]. The epithelial component is usually found within the stalk or periphery of the lesion and forms a minor portion of the tumor mass. Sometimes, there is evidence of proliferation and transition of surface basal cells to the spindle cell sarcomatous elements [7].

The sarcomatous component usually makes up the bulk of the tumor and consists of plump spindle cells, which can also be rounded and epithelioid in some regions [7]. It generally presents a fasciculated pattern which is composed of highly cellular groups of elongated bipolar cells in a parallel, interwoven alignment [6, 7]. Seldom, myxomatous, or streaming patterns can be observed, which show cells that are more stellate and pleomorphic with prominent intercellular spaces [6, 7]. A strange feature of this tumor is the relative scarcity of the carcinomatous component [7]. This creates a dilemma as the histopathologic diagnosis becomes dependent on the site of the biopsy. If it is taken from the squamous cell component, it can be misdiagnosed as carcinoma, whereas biopsies from spindle cell component tend to be misdiagnosed as sarcoma [2]. This can be the most probable explanation of arriving at a diagnosis of epithelioid sarcoma on incisional biopsy in our case. Metastatic spread of SC most frequently occurs via the lymphatic route and may consist of pure epithelial or spindle cells or of admixtures of the two histologic patterns [7].

The morphology of the spindle cells in SC cannot be just predicted by routine light microscopy but requires the use of immunohistochemistry (IHC). Cytokeratin (CK) is considered the most reliable epithelial marker but epithelial membrane antigen (EMA) and carcinoembryonic antigen (CEA) can also be useful [8]. Vishwanathan et al. in their review of 103 cases of SC observed that CK and EMA were most useful and positive in 61.3% of cases [4]. Thompson et al. in their review of 187 cases of laryngeal SC reported that 100% of cases tested expressed vimentin, with 33% demonstrating reactivity with smooth muscle actin, 15% with muscle specific actin, 5% with S-100 protein, and 2% each with desmin-D33 and desmin-DR11 [9]. IHC of the incisional biopsy in our case showed a strong cytoplasmic positivity for pancytokeratin and vimentin. EMA was also strongly positive in greater than 75% of tumor cells, whereas it was negative for S100 and desmin. Epithelial marker expression in SC decreases with a decrease in the degree of epithelial differentiation indicating that an immunopositivity although can be helpful, a negative result does not rule out the diagnosis of SC [4].

Management of SC is as tricky and controversial as its diagnosis. Wide surgical excision, with or without radical neck dissection, seems to be the most preferred and successful therapeutic modality. Radiotherapy, although considered to be ineffective by most authors, is an acceptable alternative for inoperable patients as well as for those in which the surgical margins are positive or in patients with nodal metastasis [2, 7].

Prognosis of SC is dependent on location, size, and depth of invasion of tumor, stage of disease, and the presence of any keratin staining in the spindle cells [2, 10]. SC of the oral cavity and oropharynx is potentially aggressive and tends to recur and metastasize easily [2]. Ellis and Corio reported 59 cases of oral SpCC with a 36% survival rate [11]. Olsen et al. reported 34 patients of laryngeal and hypopharyngeal SpCC with recurrence in 10 patients, mortality in 8 patients, and a 3-year survival rate of 56.8% [12]. Su et al. in their series of oral and oropharyngeal SpCC concluded that the 3-year survival rate was 27.5% [13].

## 4. Conclusion

Sarcomatoid carcinoma of the oral cavity is rare in occurrence and aggressive in nature which seems to recur and metastasize easily. A complex histogenesis makes the diagnosis of SC extremely difficult and often misleading and controversial. Diagnosis should include biopsy of the lesion from different sites to possibly include both the epithelial and sarcomatous components. A clear understanding of clinicopathologic characteristics and immunohistochemistry is indispensable for diagnosis and management of SC. Treatment should aim at controlling local and distant recurrence. Patients with deeply invasive tumors tend to have a poor prognosis, whereas those with early-stage tumors have excellent prognosis. Monophasic SC which is devoid of a classic carcinomatous component is, in some cases, indistinguishable from a sarcoma. This leads us to wonder whether every case diagnosed as SC is actually a carcinoma or true sarcoma.
